# Ultimate drivers of native biodiversity change in agricultural systems

**DOI:** 10.12688/f1000research.2-214.v1

**Published:** 2013-10-14

**Authors:** David A Norton, Nick Reid, Laura Young

**Affiliations:** 1 School of Forestry, University of Canterbury, Christchurch, 8140, New Zealand; 2 Environmental Management, School of Environmental and Rural Science, University of New England, Armidale, NSW 2351, Australia

## Abstract

The ability to address land degradation and biodiversity loss while maintaining the production of plant and animal products is a key global challenge. Biodiversity decline as a result of vegetation clearance, cultivation, grazing, pesticide and herbicide application, and plantation establishment, amongst other factors, has been widely documented in agricultural ecosystems. In this paper we identify six ultimate drivers that underlie these proximate factors and hence determine what native biodiversity occurs in modern agricultural landscapes; (1) historical legacies; (2) environmental change; (3) economy; (4) social values and awareness; (5) technology and knowledge; and (6) policy and regulation. While historical legacies and environmental change affect native biodiversity directly, all six indirectly affect biodiversity by influencing the decisions that land managers make about the way they use their land and water resources. Understanding these drivers is essential in developing strategies for sustaining native biodiversity in agricultural landscapes into the future.

## Introduction

Global land use and land cover have changed markedly over the past two centuries as a result of rapid population growth and the increasing demand for food and fibre products. As much as 75% of the Earth’s ice-free surface has been directly modified by human activities, mainly through urbanisation, timber harvesting, cultivation, and livestock grazing ^1^. These changes have resulted in land and water degradation and consequential species extinctions. Ongoing human population growth (perhaps to 9.3 billion by 2050 ^2^), including a massive increase in the size of the world’s middle class ^3^ and the concomitant increase in the consumption of meat, dairy products and luxury goods derived from animals (e.g. leather and wool), will see the demand for agricultural products increase further. This increase in demand is likely to be met through further losses of natural areas and the transformation of low-production systems to high-input farming and grazing systems. The ongoing expansion and intensification of agriculture will put more pressure on native biodiversity and the ecosystem services that biodiversity provides to humanity ^4, 5^. Sound environmental management of agriculture has never been more important for human well-being, the maintenance of global life-support systems, and the survival of planetary biodiversity.

Our concern in this paper is for the native biodiversity that evolved in an area rather than the exotic diversity that is associated with agriculture and came to that place from elsewhere ^6^. Exotic biodiversity forms the basis of most agricultural systems worldwide, including crop and livestock species, as well as the many other species that have benefitted from human activities. Maintaining the genetic diversity of exotic species used in agriculture is critical, but is not our focus. Not all changes due to agricultural management will be negative for native biodiversity: some species will prosper from changes, and remnants of some native communities will persist in a matrix of agricultural development ^7^. As a general rule however, remnant native taxa tend to be generalists and agricultural development usually results in the homogenisation of native biodiversity ^8^.

Degradation of agricultural lands and the inevitable loss of associated native biodiversity within these landscapes is one of the main consequences of the expansion in the extent and intensity of agricultural production at all scales, from local to global ^9^. Addressing land degradation and biodiversity loss while maintaining the production of plant and animal products is a key global challenge. Understanding the pressures or drivers that lead to environmental degradation and biodiversity loss is important in meeting this challenge ^10^. Indeed, it is not possible to restore degraded systems without understanding these drivers and addressing them ^11^. The importance of anthropogenic drivers of change for predicting current and future ecosystem condition was strongly emphasised in the Millennium Ecosystem Assessment ^9, 12^. Drivers are any natural or human-induced factors that directly or indirectly cause ecosystem change and biodiversity loss and include both the proximate drivers that result in actual change on the ground and the ultimate drivers that underlie these. Our focus in this article on the ultimate drivers of native biodiversity loss is motivated not by any intent to undermine the obvious importance of proximate causes of change, such as vegetation clearance, cultivation, grazing, pesticide and fertiliser application or plantation establishment, but by a desire to better understand the underlying or ultimate influences leading to day-to-day management decisions by land managers that directly affect native biodiversity.

**Figure 1.**
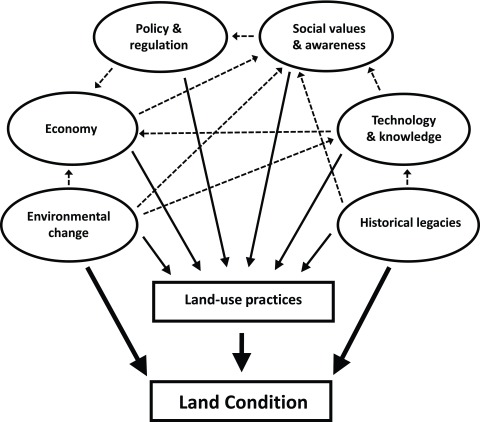
Relationship between different drivers of change and native biodiversity. While all drivers indirectly affect biodiversity through their effect on land-use practices (thin black lines), only environmental change and historical legacies directly affect biodiversity (thick black lines). Interactions (dotted lines) also occur amongst the different drivers.

In this article we outline a framework for considering ultimate drivers of biodiversity change in agricultural landscapes under six headings ( Figure 1): (1) historical legacies; (2) environmental change; (3) economy; (4) social values and awareness; (5) technology and knowledge; and (6) policy and regulation. While environmental change and historical legacies directly affect native biodiversity, all six indirectly affect biodiversity by influencing the decisions that land managers make about the way they use land and water resources. These land management decisions are critical and have a range of flow-on effects for biodiversity. We use examples from Australia and New Zealand to illustrate these underlying drivers of biodiversity change, but similar examples can be found in and applied to any agricultural system worldwide.

## Ultimate drivers

The current distribution of biodiversity in agricultural ecosystems reflects the interactions between the environment (climate, landform, soils, etc.), and the effects of past human activities (habitat creation, modification and loss, introduction of agricultural species, commensals, pesticide and fertiliser application, etc. ^7^). Legacies from these historical influences on biodiversity, together with a range of new, primarily anthropogenic, drivers will shape future biodiversity patterns directly through their influence on biodiversity (e.g. land clearance or invasive species), but most often indirectly through their influence on land and water management practices (e.g. the intensity of management inputs), which in turn have direct effects on biodiversity ^9^. The various drivers interact across multiple spatial and temporal scales ^12^ and can work synergistically. It is often synergistic effects that are of most concern for biodiversity conservation ^13– 15^. Without a clear understanding of ultimate drivers of land-use (and hence biodiversity) change, it is not possible to identify and implement appropriate strategies for biodiversity conservation, strategies that are urgently required if biodiversity decline is to be reversed over the majority of the Earth’s surface ^16– 18^.

The way that land managers respond to these drivers in terms of their farm management practices will be critical for native biodiversity, and the outcomes could either be positive or negative depending on the particular response taken. Unfortunately to date, most of the outcomes for native biodiversity have been negative. Developing a good understanding of these drivers and the ways they influence biodiversity is essential if we are to have the ability to influence the way that agricultural landscapes are managed in order to obtain better outcomes for native biodiversity. We now review each of these drivers.

## Historical legacies

Historical legacies include the many events that have occurred in the past but have an ongoing influence on both land management practices and biodiversity today. Two types of historical legacy are particularly important: effects of past land management activities on soils (e.g. erosion, salinization, compaction, pesticide application, acidification, nutrient inputs) and ongoing adjustments of remnant biotas to historical habitat destruction and fragmentation. Both of these directly affect biodiversity, while changes in soil biogeochemistry also influence farm management practices and hence biodiversity indirectly.

In some agricultural landscapes, periods of intensive land use (e.g. irrigation or fertiliser application) have resulted in fundamental changes in ecosystem attributes, especially in relation to soil properties (e.g. through salinization ^19^). These modifications have pushed ecosystems across thresholds of change that may take decades, centuries or millennia to reverse ^20^, with their effects influencing both land management practices and biodiversity for the foreseeable future. A good example of an historical legacy having a long-term effect on biodiversity is the influence of phosphorus fertiliser and exotic legume seed inputs in tableland pastures in south-eastern Australia ^21^. Elevated soil P and N levels, coupled with clearance of native timber, resulted in epidemic numbers of native scarabs, particularly Christmas beetles ( *Anoplognathus* spp., Scarabeidae). These beetles, in concert with other defoliating insects, have caused widespread dieback of pasture eucalypts, and continue to hamper establishment of eucalypts in intensively developed pasture land ^21, 22^. Elevated soil P and N are good for grass growth and the loss of tree cover has resulted in increased stocking rates because of the greater area of pasture. However, the loss of shade and shelter increases sheep mortality in extreme weather and the loss of woodland and scattered trees has caused reductions in native birds, mammals (microbats and arboreal marsupials) and lizards ^23, 24^.

Remnant biotas are still adjusting to the effects of fragmentation and the extinction debt associated with past and current habitat clearance ^25, 26^. For many species, particularly those within forested habitats, the altered environmental conditions that occur as a result of fragmentation reduce the quality of the habitat as edge effects alter forest remnant interior microclimates (e.g. making them warmer, windier and dryer ^27^). Both fragmentation itself and management practices on adjacent agricultural land can alter the disturbances that naturally occurred in these systems (e.g. fire or flooding regimes) or introduce novel disturbances such as grazing, chemical drift and weed invasion, which affect the remnant biota ^28^. Habitat loss also reduces the number of remaining individuals of a species, increasing their vulnerability to disturbance and the likelihood of local extinction, especially as isolation reduces the chance of recolonisation ^29^. At the same time, altering species distributions, both of natives and exotics, means that new species are becoming established in agricultural landscapes causing further changes to remnant biotas, the full impacts of which are yet to be felt ^30^ (see next section).

## Environmental change

While climate change and species invasion are only two of a range of environmental changes occurring globally (others include increased nitrogen deposition, land use change and intensification, and CO _2_ enrichment), they exert perhaps the strongest influence on both agriculture and biodiversity (we see land-use change as one of the proximate causes of biodiversity change). Climate change and species invasion can both directly affect biodiversity, but also have strong effects on land management practices with flow-on effects on biodiversity.

While the long-term consequences of climate change will be shifts in average rainfall and temperature patterns, it is the changes in the frequency and intensity of extreme events such as droughts, floods, wind and snow storms or frosts that are likely to be most significant for both farm production and biodiversity in the short to medium term ^31, 32^. Native biodiversity will be affected directly through changes in species distributions ^33^, including invasive species, and altering interactions between species ^34^. Biodiversity will also be affected indirectly through changing land-use patterns as farmers change their management practices to cope with climate change ^35^. While the potential direct effects of climate change on biodiversity are generally well appreciated, the indirect effects are less well understood.

**Figure 2.**
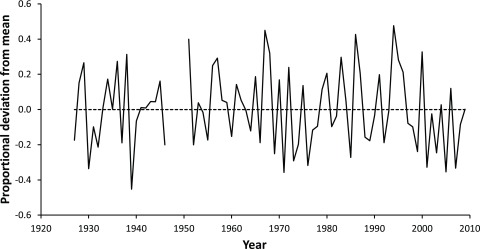
1927–2009 annual rainfall, Lake Tekapo (Station H40041), Mackenzie Basin, New Zealand (44.002 S, 170.441 E). Data (downloaded from www.cliflo.niwa.co.nz) are expressed as a proportional deviance from the 1927–2009 average value.

One example of a possible indirect effect of climate change will be through intensification of farm management practices as farmers seek to buffer themselves against the vagaries of unpredictable weather. Irrigation can be used to guarantee grass and crop growth as summer droughts become more frequent, while exotic grasses and legumes can be used to increase productivity of native dryland grasslands to better buffer these systems ^36^. For example, a succession of below-average annual rainfall years in the first decade of the 21 ^st^ century in the Mackenzie Basin in New Zealand’s eastern South Island ( Figure 2) has increased pressure on farmers to establish irrigation or plant legume crops such as lucerne as a buffer against the effects of drought. Intensification such as this has a number of flow-on effects for biodiversity, both as a result of habitat loss as remnant vegetation is cleared to accommodate more intensive farming systems ^37, 38^ and through flow-on effects to other parts of the ecosystem (e.g. eutrophication of waterways as a result of increased fertiliser inputs and irrigation, or altered river flows as a result of abstraction and regulation ^39^).

**Figure 3.**
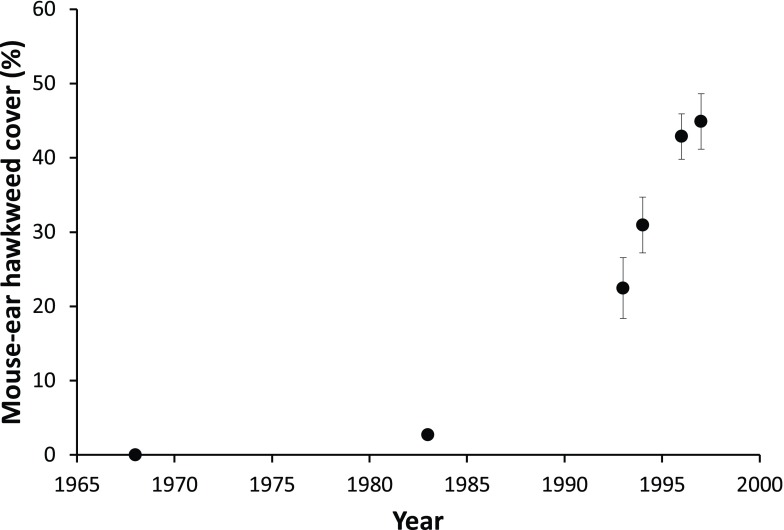
Rapid expansion of *Hieracium pilosella* in short tussock grasslands, Tara Hills Station, eastern South Island, New Zealand. (Peter Espie, unpublished data with permission).

Invasive species are another important part of environmental change and have the potential to be a key driver of the future condition of biodiversity. In particular, a vast pool of introduced species, especially plants, occur in gardens or as individuals in the wild but do not yet have an obvious impact on either agriculture or native biodiversity ^40^. These species may never become a problem, but equally, should environmental conditions or land management practices change, or if they simply cross an abundance threshold, then they could have major impacts in the future. It is difficult to predict when or why a species will start to rapidly expand, but it is clear that for many species this might not occur for years or even decades after the species is first naturalised ^41^. An example of this is the European daisy, mouse-ear hawkweed ( *Hieracium pilosella*, Asteraceae), which was present in New Zealand as early as 1878 (it most likely arrived as a contaminant in grass seed) but wasn’t recognised as a problem until the 1970s when it started to rapidly expand across a wide range of sites in the eastern South Island ( Figure 3). This species has become a major pest in the rain-shadow mountains of this region where it has invaded grasslands, significantly reducing both livestock feed and biodiversity values through outcompeting other plant species for water and nutrients ^42^.

## Economy

The economy (from local to global) is perhaps the biggest driver of agricultural decision making, and hence of change in native biodiversity. Except in subsistence systems, farmers are exposed to the vagaries of international markets including the price of farm inputs (especially fuel and fertiliser), changing consumer preferences and demands, the availability of substitutes (e.g. synthetics for natural fibres) and cheap imports, and the effects of national and global economies (e.g. cycles of recessions and booms) on key farm costs such as mortgage interest rates ^43^. Reduced farm incomes also flow on through taxation to the funding available for conservation initiatives at a regional and national government level. While the state of the global economy does not have a direct effect on biodiversity, it has two main consequences that can have marked flow-on effects for biodiversity through effects on farm management: (1) changes in the type of farming operation that is undertaken in response to economic conditions (e.g. the shift towards dairy farming in New Zealand in response to high global dairy prices), and (2) changes in the profitability of the farming operation itself, thus affecting both what the farmer can afford to do (e.g. weed or pest control) and indirectly what government can afford to do in terms of biodiversity conservation (e.g. through financial incentives to farmers to undertake conservation work) through lower taxes.

If prices squeeze profit margins ^44, 45^, farmers might seek to increase economies of scale in their operations, which might affect biodiversity, for example, through an increase in the size of individual paddocks with a resultant loss of remnant areas of biodiversity (as already occurs when large pivot irrigators are installed ^37^). Alternatively farmers might be forced to scale back their operations and especially the use of inputs such as fertiliser, which could be good for biodiversity; this occurred in New Zealand when widespread farm subsidies were removed in the 1980s ^46^. Another conundrum that can occur is that declines in farm returns can be good for biodiversity as farmers reduce their investment in on-farm improvements enabling native plants and animals to reoccupy areas they had been excluded from by agricultural practices. However, good financial returns could be either positive or negative for biodiversity: positive in that farmers have more disposable income to spend on non-essential activities such as biodiversity conservation, but negative in that in other situations farmers might choose to intensify their management and increase productivity to take greater advantage of higher returns.

One of the key causes of increased agricultural production over the last 50 years has been the increased use of fertiliser, especially nitrogen-based fertilisers ^4^. Fertiliser production is dependent on both supply (especially for fertilisers based on mining naturally occurring deposits, such as phosphorus) and the energy costs of fertiliser production. Spikes in global fertiliser prices in the mid-1970s and late-2000s led to reductions in fertiliser application in New Zealand as farmers had to trade off productivity gains against the increased cost of fertiliser. Cheap fertiliser enables farmers to intensify their management with obvious impacts on biodiversity, while high fertiliser prices generally mean that fertiliser use declines. It is clear that the supply of both fertiliser and fuel is not finite ^47^, but the consequences for biodiversity of rapidly increasing prices of both are unclear. Technology may well provide alternatives to traditional fertilisers or alternative ways to retain key nutrients within agricultural systems might be developed. For example, various types of holistic management aim to reduce or even eliminate the use of inputs such as fertilisers by adopting alternative grazing systems and through the increased use of other nutrient sources such as N-fixing trees. Holistic management (e.g. http://holisticmanagement.org/) and regenerative agriculture (e.g. http://regenag.com/web/) are two examples of these types of approaches. Both are likely to be positive for biodiversity as they encourage a more environmentally sympathetic approach to land management and are attractive to farmers who already have a personal empathy for the environment. High fertiliser prices might force farmers to effectively ‘abandon’ some parts of their properties or alternatively farmers might choose to increase their stocking rates across their whole property to effectively ‘mine’ the resources present in order to maintain a certain level of profit in the face of rising prices.

## Social values and awareness

The way we value biodiversity and agriculture has a fundamentally important influence on what happens to native biodiversity in agricultural landscapes. This is particularly evident with respect to the way in which society’s valuation of agricultural production versus biodiversity conservation has changed through time. As our values and awareness has changed, so too have the ways that we approach land management and hence its impacts on native biodiversity.

When European settlers first colonised Australia and New Zealand, elements of the native biodiversity were seen as a hindrance to ‘good’ land management ^48^. The new settlers struggled to understand the local environment while land management approaches were biased by the European agricultural tradition and ‘what worked best’. Few people at that time believed that indigenous ecosystems, especially timbered areas, held any value beyond that of the timber and grass they supported, or the land on which they grew. In fact woody vegetation and wetlands were widely seen as a limitation to ‘progress’. Settlers actively sought to impose a European mantle across the land by clearing timber, draining wetlands, planting Northern Hemisphere species, and introducing livestock ^48^. Government policy at the time in both Australia and New Zealand actively promoted this through the setting of land clearance targets that needed to be met before land ownership could be gained (e.g. the Robertson Land Acts in the 1870s in New South Wales). Links between forest cover and soil and water values were not appreciated, while stock numbers in rangelands reached densities never matched subsequently as flocks of sheep in particular exploited the available forage ^49^.

**Figure 4.**
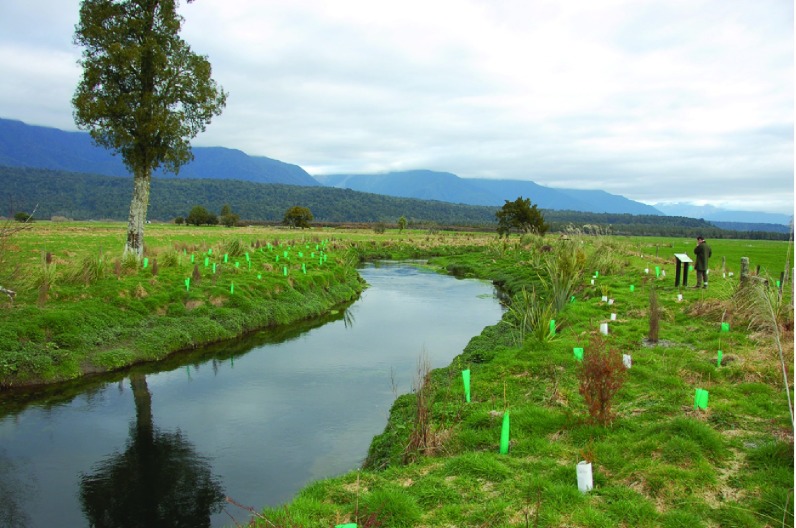
Riparian fencing and recent restoration plantings, Westland, New Zealand. (Photo: David Norton with permission).

Values and awareness have changed over time, both in Australia where aridity and frequent droughts made it difficult to impose the traditional European agricultural model over most of the continent, and in New Zealand where it was realised that a lack of trees resulted in severe soil erosion and loss of the productive potential of the land that farmers were seeking to utilise ^50^. More recently the importance of wetlands as filters to reduce the impacts of agricultural pollutants has also been recognised ^51^ ( Figure 4). Soil and water conservation became necessary and new land management practices were developed to cope with the profound changes that resulted from European-style farming. Contour banking of sloping croplands in Australia ^52^ and tree planting with exotic species in New Zealand were undertaken to reduce soil erosion ^50^. While the importance of setting aside natural areas for biodiversity conservation has been long recognised (e.g. the national park and reserve systems in Australia and New Zealand date back to the late nineteenth century), the intrinsic value of native biodiversity in agricultural landscapes above and beyond its utilitarian value for grazing and timber, and especially its role in providing key ecosystem services (e.g. pollination) has only been recognised in recent decades. Policy and regulation in this area still lags behind the preservation policies that underpin public conservation lands and a shift of emphasis is urgently needed within land management, research and policy to address this imbalance ^53^.

## Technology and knowledge

**Figure 5.**
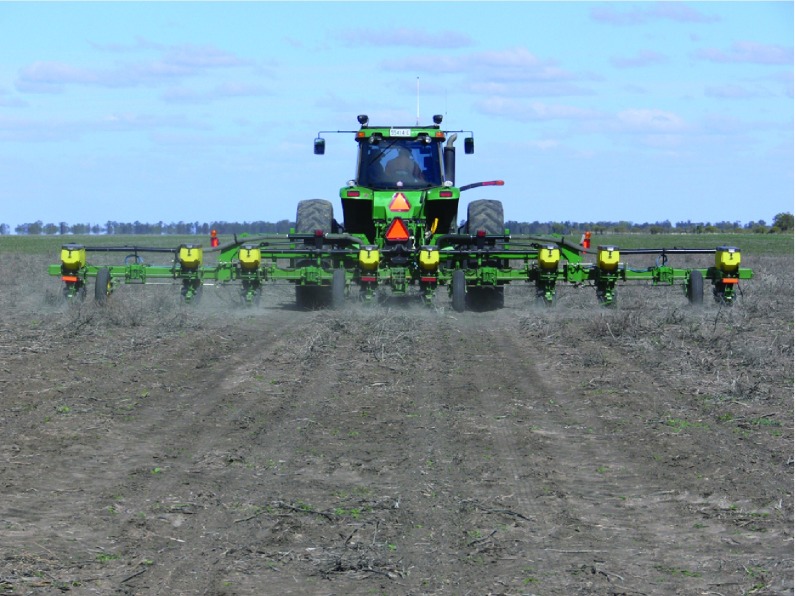
Cultivating heavy cracking clay soils on inland floodplains, New South Wales, Australia. (Photo: Leah MacKinnon with permission).

Technology and knowledge are also important drivers of change in agricultural systems. The availability and affordability of new technology has revolutionised agriculture over the last 50 years. Technological advances in plant breeding and genetic engineering, the use of new herbicides and pesticides, the development of larger, more powerful machinery (tractors, irrigators, etc.), and the application of precision agriculture have all enabled farmers to produce more food and fibre at lower cost, and to move into environments that previously were not able to be farmed (e.g. the heavy soils on alluvial floodplains that early post-war machinery could not cultivate; Figure 5). The development of new genotypes through plant breeding programmes and the incorporation of genetic engineering technology to increase disease resistance and productivity and facilitate weed control has also allowed farmers to increase output of food and fibre production ^4, 54^.

Technology is both positive and negative for biodiversity: positive in that many of the new technological advances have enabled farmers to better target their management interventions, thus sparing areas with higher biodiversity values (e.g. through the use of GPS to guide application of fertiliser or herbicide) and in being able to increase productivity in one part of the farm and spare or rest other areas and avoiding adjacent natural areas such as wetlands ^55^. Technological advances have also enabled the application of more environmentally sustainable management practices such as direct drilling as opposed to ploughing ^56^, especially where native pastures can be direct drilled with production species without affecting the native biodiversity present (called pasture-cropping ^57^). However, the advent of new technology has also enabled farmers to farm new areas, especially areas that might otherwise have been marginal or too difficult to crop, and to increase the size of individual management units (e.g. paddocks) and farms in order to capitalise on economies of scale, further increasing impacts on native biodiversity through homogenising farmscapes ^8^.

Some technological advances have been both positive and negative. For example, direct drilling enhances soil condition by removing the need for cultivation, but the increased use of herbicides and spray drift may affect adjacent native ecosystems. Genetic engineering has allowed farmers to grow crops that are resistant to glyphosate and certain insect herbivores. Genetically modified glyphosate-resistant crops allow over-spraying with herbicides to control weeds in the crop, both with adverse effects due to herbicide drift into adjacent native vegetation and concomitant loss of habitat of native fauna ^58^. In addition, increasing use of glyphosate has fostered selection of glyphosate-resistant weeds, which are now widespread across Australian irrigated farming districts ^59^. However, the development through genetic engineering of *Bacillus thuringiensis* (Bacillaceae) insect resistance in commercial crops such as rice and cotton has been positive for native biodiversity, as it requires less pesticide application, which may well have benefits for native invertebrates ^60, 61^. However, genetic engineering has not yet been able to address abiotic stressors of plant productivity such as drought ^62^, although Monsanto have recently developed a genetically modified form of maize including a gene that enables plants to decrease their soil water absorption rate under dry conditions ^63^. Such developments could potentially provide farmers with another tool for improving resilience in the face of climate change, although traditional plant breeding at this stage appears just as effective as genetic modification ^63^.

Knowledge is a key driver of change in agricultural systems, both in relation to farming practices and biodiversity. Knowledge feeds into social values and awareness as well as having a direct impact on the way land is managed and hence on biodiversity. Much of the impact of past agricultural practices on biodiversity has occurred because farmers (and land managers more generally) have not had the relevant information to enable them to realise the adverse consequences of their activities. The recent recognition of the importance of biodiversity conservation in agricultural landscapes, especially in ‘new’ countries like Australia and New Zealand, has limited the attention that biologists have given to these areas. Several studies have highlighted the lack of ecological research undertaken in agricultural landscapes ^64, 65^. For example, a survey of New Zealand ecological literature published over the period 1968–1997, found that 65% of articles focused exclusively on protected areas while 18% focused exclusively on non-protected areas (mainly agricultural and plantation forest landscapes), despite non-protected areas accounting for nearly 70% of the total land area ^66^. While native biodiversity in agricultural landscapes has received more attention in recent years ^7^, there are still substantial gaps in our knowledge in terms of just what native biodiversity is present there, how this biodiversity is impacted by agricultural practices, and what ecosystem services this biodiversity provides to agricultural production specifically and society more generally.

## Policy and regulation

Policy and regulation develop in part from social value systems, but in many ways are not in tune with current societal values or awareness of issues. This occurs because the politicians and bureaucrats who develop policy are often not willing to tackle current societal issues (as is clearly evidenced by the gun debate in the USA), perhaps because of the pressures associated with short election cycles, and because the vast majority of society simply does not have any idea of the diversity of issues that are regulated and hence is not involved in debates about them. This leaves policy making and regulation in the hands of a very small group of politicians, bureaucrats and lobby groups. In addition, historical legacies of past policy, including the case law associated with legislation, drive ongoing policy development. Notwithstanding this, policy and regulation exert a very powerful influence on land management decision making ^67^, with both positive and negative outcomes for biodiversity. Policy influences decision making at every level from international to national and regional levels. Protectionist trade tariffs are one example where international policy can directly influence the types of decisions made by farmers ^30^, for example on the type of farming that is undertaken. The removal of trade barriers is also likely to further facilitate the flow of invasive species as trade increases facilitating these species in reaching new areas through increased dispersal opportunities ^68^.

Domestic policy and associated regulation also have a marked impact on a farmer’s decision making. Regulation can be particularly poor in mitigating the many small incremental actions of multiple actors. This is illustrated by vegetation clearance rules that are widely used to restrict the amount of native vegetation cleared in Australia and New Zealand ( Supplementary material). However, if every farmer in a region cleared what was legally possible under exemptions associated with such rules, biodiversity would continue to decline. Furthermore, some farmers look at vegetation clearance rules as a cost of business, with the fine or penalty associated with breaking the rule being trivial compared to the potential profits that would result from utilising newly cleared areas. Blunt regulatory instruments are weak at controlling gradual land-use intensification. (e.g. the loss of palatable native plants from fertilised and over-sown pastures subjected to increased stocking rates), nor do they deal with the habitat loss resulting from species invasions. Some policy can have unintended positive outcomes for biodiversity. In New Zealand, a change in government in the early 1980s resulted in the removal of a range of farm subsidies. As a result of this, fertiliser price increased locally, even though global fertiliser prices were low, resulting in farmers using less fertiliser and in some cases abandoning less productive land with many of these areas now regenerating back into native forest ^69, 70^.

## Conclusions

The effects of different drivers on farmers’ decision making and hence on land management activities and native biodiversity are complex and often interrelated. The following example illustrates how two of these drivers can affect native biodiversity in a livestock production system through their influence on farm management practice. Both environmental change and the international market place can place substantial pressure on the profitability of a farm business, especially where financial commitments such as mortgage payments are an issue. In the case of environmental change, this can be through changes in the frequency and intensity of extreme climatic events such as droughts, heatwaves, floods, windstorms or frosts that are significant for farm production ^31, 32^. In the case of international markets, factors such as increasing costs of external inputs such as fuel and fertiliser coupled with fluctuating returns for farm products are critical. One response to these types of pressure is to intensify farm management practices in order to buffer the farm business against the vagaries of unpredictable weather or markets. Intensification can involve one or more management actions (the proximate drivers of change), including increasing the carrying capacity of existing pastures (e.g. through cultivation or topdressing with seed and fertiliser), bringing new land into production through native vegetation clearance, or by overgrazing the existing forage base (as a short-term strategy to cope with immediate financial challenge). All of these can result in land degradation and loss of native biodiversity.

Agricultural systems are of course very diverse and each driver will have a different effect depending on the nature of the particular system including the type of farming system (grazing, cropping, irrigated, rain-fed, with or without woody vegetation, etc.), the productivity of the system (annual yield of grain, milk solids, meat, etc.), and the amount and distribution of native biodiversity present. The potential to intensify farm management for whatever reason is the primary cause of native biodiversity vulnerability ^30^, as intensification almost always results in the loss of native species, both where this involves clearing areas of remnant native vegetation or through transformation of current farmed areas from low-producing to high-producing states. In contrast, native biodiversity in systems that have relatively little potential for intensification, either because they have already been intensively developed (e.g. irrigated arable or dairy farming systems) or because productivity is limiting and cannot be readily enhanced (e.g. rangeland systems lacking water), is likely to be less vulnerable.

An appreciation of the drivers of biodiversity change within agricultural ecosystems is important as it enables us to better plan management activities in order to achieve desired outcomes for native biodiversity. Distinguishing between direct and indirect drivers is particularly important, as it is the indirect or ultimate drivers that are likely to have the greatest influence on future biodiversity given that the primary use of these ecosystems is for the production of food and fibre. The drivers discussed here are ultimately responsible for most impacts on native biodiversity in agricultural systems, both directly but especially through their strong influence on farm management practice. Management in turn dictates the nature and habitat qualities of the production matrix and shifts remnants of native ecosystems into structural and compositional states further from their historical condition ^71^. These changes are likely to require novel approaches for the long-term management of native biodiversity ^72^ as well as an explicit evaluation of the value of this biodiversity, both for itself (intrinsic value) and for the ecosystem services that it provides to farm production and to society more generally.

In order to address the effects of the different drivers of native biodiversity in agricultural landscapes, mechanisms are required to encourage farmers to incorporate management of native species into overall farm management practices. In some cases, all that is required is to facilitate the farmer to continue with their existing management approach. However, more often mechanisms are required to assist farmers to change their management or adopt new practices that are more sympathetic to native biodiversity ^7^. In selecting the most appropriate approach for doing this, it is necessary to consider the relative levels of public (external) and private (internal) benefits of a given land management practice ^73^ and to ensure that these are appropriately accounted for. Simply putting in place regulations that limit carrying capacity or vegetation clearance do not necessarily address the underlying (ultimate) reasons driving management decisions. In fact, poorly thought through regulations can actually result in unintended or perverse outcomes (e.g. proposed vegetation clearance rules resulting in a flurry of clearance before regulations are enacted). Therefore, in order to address the indirect effects of the ultimate drivers of biodiversity change considered here, other approaches need to be considered. Such approaches could better align on-farm decision making with positive biodiversity outcomes, for example, by offering financial incentives to farmers to retain native vegetation and provide ecosystem services such as clean water and carbon sequestration ^74, 75^.

## Supplementary material

Examples of a vegetation clearance rules in three New Zealand district plans (local government land planning statutes).

### Waikato District Plan rule 25.43.1

( www.waikatodistrict.govt.nz/CMSFiles/ca/cab18ac7-cba6-49e1-888b-4c9d5efdc1cb.pdf)

Vegetation clearance of indigenous vegetation or habitat of indigenous fauna in a Landscape Policy Area or Conservation Policy Area is a permitted activity if:

(a) it does not exceed 1000m ^2^ or 1% of contiguous indigenous vegetation or habitat of indigenous fauna, whichever is the lesser, per contiguous area per site in any 3-year period, and is limited to:

    (i) a building platform for a permitted or approved building, or structure(s) or access, or

    (ii) gathering of plants in accordance with Maori custom and values, or

(b) it does not exceed 3000m ^2^ or 3% of contiguous indigenous vegetation or habitat of indigenous fauna, whichever is the lesser, per contiguous area per site in any 3-year period, and is limited to:

    (i) maintaining or reinstating productive pasture and productive forests and maintenance of tracks and fences through the removal of manuka and/or kanuka and/or treeferns that are more than 10m from a water body and less than 15 years old or less than 5m in height and any under-storey under such manuka or kanuka or treeferns growing on land that was previously in productive use.

### Hurunui District Plan rule A7.2.1

( www.hurunui.govt.nz/assets/Documents/District%20Plan/2-A-07%20Natural%20Environment.pdf)

(d) Wetlands

There shall be no clearance or cultivation or damage by deposition of material or infilling of indigenous vegetation in any wetland exceeding 1000 square metres in area.

(e) Clearance of indigenous vegetation

    (i) No clearance of indigenous vegetation not already significantly modified by any farming practice, other than as provided for in

    - Section A2 – Landscape

    - Section A7 – Natural Environment

    - Section B2 – Coastal Environment

    - Section B3 – Hurunui Lakes Area

    shall be permitted of greater than 1 hectare over a 5-year period on any separate certificate of title.

### Mackenzie District Plan rule 12.1.1g

( www.mackenzie.govt.nz/Site/Documents_and_policy/key_documents/district_plan.aspx)

Short Tussock Grasslands

On each of the individual farm properties existing in the Mackenzie Basin Map as at 1 January 2002 in any continuous period of five years there shall be no clearance including cultivation above the following thresholds of short tussock grasslands, consisting of silver or blue ( *Poa* species), or *Elymus solandri*, or fescue tussock where tussocks exceed 15% canopy cover:

    (i) 40 hectares or less – Permitted Activity

    (ii) Greater than 40 hectares – Discretionary Activity.
