# Supplementary material for: Ultimate drivers of native biodiversity change in agricultural systems
Source: F1000Res. 2013 Oct 14;2:214. [Version 1] doi: 10.12688/f1000research.2-214.v1 (PMC4722685; doi:10.12688/f1000research.2-214.v1)
Supplement: Peer review history

# Peer review: Referee response for version 1

**DOI:** 10.5256/f1000research.2650.r3305

**Published:** 2014-02-10

**Authors:** Rachel Standish

**Affiliations:** 1 School of Plant Biology, University of Western Australia, Crawley, Australia

As the title of this paper suggests, it reviews the ultimate drivers of biodiversity change in agricultural systems using examples from eastern Australia and New Zealand. The authors are well-respected for their contribution to ecological research, and particularly for informing the options for the conservation of biological diversity in these landscapes. Therefore, the review is as I’d expect it to be—authoritative and conceptually sound. In particular, Figure 1 will be a useful framework to prompt discussions among land managers and other stakeholders. An example of a decision informed by the framework would be a valuable addition to the paper.

More generally, however, the review paints a fairly pessimistic view for biodiversity conservation in agricultural landscapes and reading it left me wanting to know more about the strategies mentioned in the final sentence of the abstract. The authors deliberately reviewed the ultimate drivers of biodiversity change, because unlike proximate drivers, these can be managed—but how? For example, what ecologists might do to fill the knowledge gaps? How might the emerging carbon market be developed to benefit biodiversity and farmers in agricultural landscapes of Australia and New Zealand ^1^? What are some other examples of potential win-win farming enterprises? How might trade-offs among ecosystem services, including both ecological and socio-economic perspectives, affect outcomes for biodiversity in these landscapes?  There is some evidence that traditional, as opposed to novel, approaches such as fencing to exclude livestock and unassisted regeneration of abandoned farmlands can help biodiversity conservation in agricultural landscapes ^2^. What are the incentives for farmers to adopt such practices?

These are my other, more minor points:

1. Historical legacies are particularly important in the ancient landscapes of south-western Australia ^3^ ^4^. Land-use legacies combine with seed dispersal and recruitment limitations to form ecological thresholds to recovery ^5^ ^6^. The socio-economic drivers of land-use change are also documented for this region ^7^ ^8^.
2. I agree that land-use intensification generally results in the loss of native species, but what about the options for biodiversity conservation at larger scales if intensification is paired with land-sparing? Indeed, the current global debate about land-sparing vs sharing would be relevant to mention here.
3. Nested within social values and awareness are the management goals we as a society set for biodiversity conservation. I think this section of the paper could introduce some of the novel approaches mentioned in the discussion. For example, where and when might it be appropriate to consider novel ecosystems as goals for management in place of historic ecosystems?
4. While it is probably true that agricultural landscapes are understudied compared with other landscapes in Australia as well as New Zealand, this point seems to detract from the arguably more valid point that there’s decades worth of research from which to base good decisions ^9^. 

Thank you for the invitation to review this paper.  I enjoyed thinking about it.

I have read this submission. I believe that I have an appropriate level of expertise to confirm that it is of an acceptable scientific standard.

## References

[1] StandishRJHulveyKB: Co-benefits of planting species mixes in carbon projects. *Ecological Management & Restoration*.2014;15(1) : 214 26-29 10.1111/emr.12084 Reference source

[2] ProberSMStandishRJWiehlG: After the fence: vegetation and topsoil conditions in grazed, fenced, and benchmark eucalypt woodlands of fragmented agricultural landscapes. *Australian Journal of Botany*.2011;59(4) : 214 369-381 10.1071/BT11026 Reference source

[3] StandishRJCramerVAHobbsRJKobrynHT: Legacy of land-use evident in soils of Western Australia's wheatbelt. *Plant and Soil*.2006;280(1-2) : 214 189-207 10.1007/s11104-005-2855-6 Reference source

[4] StandishRJCramerVAHobbsRJ: Land-use legacy and the persistence of invasive Avena barbata on abandoned farmland. *Journal of Applied Ecology*.2008;45(6) : 214 1576-1583 10.1111/j.1365-2664.2008.01558.x Reference source

[5] StandishRJCramerVAWildSLHobbsRJ: Seed dispersal and recruitment limitation are barriers to native recolonization of old-fields in western Australia. *Journal of Applied Ecology*.2007;44(2) : 214 435-445 10.1111/j.1365-2664.2006.01262.x Reference source

[6] CramerVAHobbsRJStandishRJ: What's new about old fields? Land abandonment and ecosystem assembly. *Trends Ecol Evol*.2008;23(2) : 214 104-112 10.1016/j.tree.2007.10.005 Reference source 18191278

[7] AllisonHEHobbsRJ: Resilience, Adaptive Capacity, and the "Lock-in Trap" of the Western Australian Agriculture Region. *Ecology and Society*.2004;9(1) : Reference source

[8] SmithFP: Who's planting what, where and why - and who's paying?: An analysis of farmland revegetation in the central wheatbelt of Western Australia. *Landscape and Urban Planning*.2008;86(1) : 214 66-78 10.1016/j.landurbplan.2007.12.008 Reference source

[9] ProberSMSmithFP: Enhancing biodiversity persistence in intensively used agricultural landscapes: A synthesis of 30 years of research in the Western Australian wheatbelt. *Agriculture, Ecosystems & Environment*.2009;132(3-4) :173-191 Reference source

# Peer review: Referee response for version 1

**DOI:** 10.5256/f1000research.2650.r2330

**Published:** 2013-12-20

**Authors:** Bruce Clarkson

**Affiliations:** 1 Environmental Research Institute, University of Waikato, Hamilton, New Zealand

This is an insightful opinion piece and deserving of publication. 

The introduction in particular is succinct and to the point. The key scene setting statement for me is: “As a general rule however, remnant taxa tend to be generalists and agricultural development usually results in homogenisation of native biodiversity”. 

I have twice in my career said as much in "Conservation at the end of the century: challenges, crises and contradictions" ^1^ and Lee, Meurk & Clarkson, 2008 - “only a small generalist subset of the indigenous flora and fauna can persist in, or recolonise, intensified agricultural landscapes” ^2^.

The statement is critical because it begs the question is it even realistic to think that indigenous biodiversity can ever be adequately represented within essentially intensive productive agricultural lands?

My only criticisms (minor) of the paper are around areas where I would argue for a different or slightly different emphasis. We all have different backgrounds and so depending on our experiences there are likely to be questions of emphasis. I deal with these in turn below:

Page 5: left hand column last sentence regarding good financial returns; the non-essential activities are ***perceived ***non-essential activities, which if an ecosystems services or natural capital accounting model was in place may not be viewed as non-essential.

Page 5: right hand column last paragraph; the European settler approach was also a pragmatic one, driven (economic driver) by the products required for export to the home country. As the historian WH Oliver has succinctly noted, this agricultural land use was driven by the imperative to survive in a new land by producing trade goods demanded by colonial masters across the sea, and the land has disproportionately suffered as a result. The settlers were just responding to a market signal, and there was no other market?

Page 6: left hand column last sentence; seems that this is one place that the lack of comment policy and regulatory incentives, and the development of self-organised responses to the problem e.g. QE II Trust and Landcare groups, could have been addressed?  Additionally the Lee *et al.,* 2008 paper ^2^, in response to the Moller *et al*., 2008 article ^3^, took a view that regulation needed more emphasis than incentive, but even that may still be insufficient to stop an extinction pathway for indigenous biodiversity under agricultural intensification?

Page 7: left column top para; it's not just new technology that enables development of previously marginal lands, but ready access and/or cheaper availability of existing technology e.g. the swamp forests on the Egmont ringplain habitat reduction by modern drain digger vehicles.

Page 7: left column last para; the claim that farmers lacked the relevant information led to an inability to realise adverse consequences of their activities seems too simplistic. What about barriers to adoption, the need for first adopters, lack of awareness, failure to observe nature, ignorance, bloody mindedness, deliberate ignoring of warnings. There are multiple reasons for a lack of realisation?

Page 7: Policy and regulation section takes a very narrow view focusing on vegetation clearance rules by themselves, but without any quantification of how clearance rules may have slowed clearance or how when coupled with incentives e.g. covenanting incentives linked to SNA scheduling to retain has had a positive impact on the Egmont ringplain.

Page 8 Conclusions: I reiterate here the lack of mention of policy and regulatory incentives and the development of self-organised responses to the problem e.g. QE II Trust and Landcare groups. Making statements like "..mechanisms are required to encourage farmers…" or "simply putting in place regulations… do not necessarily address…"or "…other approaches need to be considered" just begs a lot a questions and a single answer by offering financial incentives is not nuanced enough: what types of incentives?  Environmental, Biodiversity or Carbon Credits, tax relief? Rates relief? These are positive financial incentives but what about negative financial incentives, such as trading barriers or forms of environmental accreditation etc. Is it other approaches or just a wider diversity of approaches including regulation an positive and negative financial incentives?And who is responsible for doing this - Central Government and Local Government?

And if all of this new approach is put in place, is the prognosis for native biodiversity in agricultural systems: very optimistic, reservedly optimistic or not optimistic?

A valuable contribution; thanks for the opportunity to comment.

I have read this submission. I believe that I have an appropriate level of expertise to confirm that it is of an acceptable scientific standard.

## References

[1] ClarksonB: Conservation at the end of the century: challenges, crises and contradictions. In: New Zealand Botanical Society Conference. *New Zealand Botany at the end of the Millennium*.17-19th June 1999;

[2] LeeWGMeurkCDClarksonBD: Agricultural intensification: whither indigenous biodiversity?. *New Zeal J Agr Res*.2008;51(4) : 214 457-460 10.1080/00288230809510475 Reference source

[3] MollerHMacLeodCJHaggertyJRosinCBlackwellGPerleyCMeadowsSWellerFGradwohlM: Intensification of New Zealand agriculture: Implications for biodiversity. *New Zeal J Agr Res*.2008;51(3) : 214 253-263 10.1080/00288230809510453 Reference source
